# miR-150 regulates endothelial progenitor cell differentiation via Akt and promotes thrombus resolution

**DOI:** 10.1186/s13287-020-01871-9

**Published:** 2020-08-12

**Authors:** Xiaolong Du, Nan Hu, Huiying Yu, Lei Hong, Feng Ran, Dian Huang, Min Zhou, Chenglong Li, Xiaoqiang Li

**Affiliations:** 1 Department of Vascular Surgery, Nanjing Drum Tower Hospital, The Affiliated Hospital of Nanjing University Medical School, Nanjing, 210008 China; 2 Department of Vascular Surgery, The Second Affiliated Hospital to Soochow University, Soochow University, Suzhou, 215000 China; 3 Department of Vascular Surgery, Jining No. 1 People’s Hospital, Jining Medical College, Jining, 272000 China

**Keywords:** miR-150, Endothelial progenitor cells, Differentiation, Akt/FOXO1 pathway, Deep venous thrombosis

## Abstract

**Background:**

Deep venous thrombosis (DVT) constitutes a major global disease burden. Endothelial progenitor cells (EPCs) have been described in association with recanalization of venous thrombus. Furthermore, emerging evidence suggests microRNAs are involved in this progression. The goal of this study was to investigate the influence of miR-150 on the behavior of EPCs and its potential contribution in venous thrombosis resolution.

**Methods:**

We isolated and cultured EPCs from healthy adults. Next, early EPCs or endothelial colony-forming cells (ECFCs or late EPCs) were transfected with miR-150 agomir and antagomir. Gene expression profiles, proliferation, cytokine secretion, and angiogenic capacity of early EPCs and ECFCs were examined. The effects of miR-150 on c-Myb expression and Akt/FOXO1 signaling were also evaluated. Furthermore, a rat model of venous thrombosis was constructed to determine the in vivo function of EPCs.

**Results:**

Our results showed that miR-150 overexpression in early EPCs significantly promoted differentiation to ECFCs and contributed to proliferation and tube formation. However, suppression of miR-150 in late EPCs inhibited proliferation and tube formation. Moreover, we identified that this progression is regulated by inhibition of c-Myb and activation of the Akt/FOXO1 pathway. Our findings also showed that miR-150 led to the enhanced resolution ability of EPCs in a rat venous thrombosis model.

**Conclusions:**

In this study, we present a novel mechanism of miRNA-mediated regulation of EPCs and Akt activation in thrombus resolution.

## Background

Deep venous thrombosis (DVT) is a major global burden with about 10 million cases occurring every year [1]. DVT is associated with substantial morbidity and mortality with 40% of patients diagnosed with proximal DVT demonstrating associated pulmonary embolism (PE) [2]. Approximately 20–50% of patients with symptomatic DVT will develop post-thrombotic syndrome (PTS), which is characterized by chronic pain, intractable edema, skin alterations, and leg ulcer [3]. Anticoagulation remains the primary treatment for DVT; however, up to 50% of proximal DVT patients will develop PTS despite optimal anticoagulant therapy [4]. Therefore, it is critical to develop additional safe and effective therapies for DVT.

Endothelial progenitor cells (EPCs) are precursors of mature endothelial cells and heavily participate in post-natal neovascularization [5]. Evidence suggests that the number and function of EPCs contribute to endothelial repair and angiogenesis of damaged blood vessels [6]. Many studies indicate that EPCs are not a single cell type, but rather two types called early EPCs (eEPCs) and endothelial colony-forming cells (ECFCs or late EPCs) [7, 8]. EPCs are defined primarily by phenotype and biological properties. Early EPCs have a short lifespan (3–4 weeks) and express CD133+CD45+, while ECFCs live longer, proliferate rapidly, and are CD31+KDR+. In addition, different angiogenic properties between these two EPC subpopulations have been documented. eEPCs showed limited proliferation capacity while ECFCs are highly incorporated into vascular networks [9]. By way of contrast, eEPCs, but not ECFCs, indirectly augment tubulogenesis even when physically separated by a transwell membrane, which implies that the effect is via a paracrine mechanism [9–11]. Given their different roles and functions in angiogenesis and paracrine, interest in understanding and manipulating EPCs for therapeutic purposes has increased.

MicroRNAs (miR), a class of ~ 22-nt non-coding RNAs, participate in various biological processes including cell proliferation, differentiation, and apoptosis [12–14]. These single-strand RNA base pairs with perfect or imperfect complementary sequences located in 3′ untranslated regions (3′UTRs) of target genes, leading to target mRNA degradation or reduction in protein translation [15, 16]. Furthermore, miRNAs are likely important in multiple biological processes of EPCs [17]. miR-130a has been shown to be involved in autophagy regulation in EPCs through Runx3 [18]. miR-34a appears to participate in modulating differentiation in EPCs via targeting Forkhead box j2 [19]. In addition, our previous research showed that miR-483-3p upregulation and let-7e-5p downregulation contributed to EPC (most of which are ECFCs) function in vitro and improved thrombus resolution in vivo [6, 20].

miR-150 has been shown to be selectively expressed in mature B and T cells and is important for immune cell differentiation and activation [21]. Evidence suggests miR-150 and miR-99a cooperatively repressed the expression of the Th17-promoting factor mTOR to stimulate regulatory T cell differentiation [22]. Sun and colleagues [23] established that miR-150 regulated proliferation and differentiation in terminal erythropoiesis. They found that forced expression of miR-150 suppressed erythroid proliferation by apoptotic induction and cell cycle blockade while miR-150 inhibition promoted terminal erythropoiesis. We recently identified miR-150’s ability to regulate EPC motility via targeting c-Myb and enhancing EPC homing and thrombus resolution in vivo [24]. However, the underlying mechanism of miR-150 regulation of EPC biological processes remains unclear.

In the present study, we discovered that miR-150 was significantly associated with EPC differentiation. Moreover, miR-150 activated Akt/FOXO1 signaling and promoted c-Myb suppression via direct targeting of 3′UTRs. In summary, our results suggest that miR-150 is important in EPC differentiation and may represent a novel therapeutic strategy in DVT.

## Methods

### Isolation and culture of EPCs

Peripheral blood (80 ml) was collected from healthy adult volunteers under informed consent. All protocols were approved by the Institutional Review Board of Nanjing Drum Tower Hospital, the Affiliated Hospital of Nanjing University Medical School. EPCs were isolated and characterized according to previous methods [25]. Peripheral blood mononuclear cells (PBMCs) were isolated from the blood by density gradient centrifugation using Histopaque-1077 (Sigma-Aldrich, MO, USA), and 2 × 10^7^ cells/cm^2^ were seeded on fibronectin-coated 6-multiwell dishes (5 μg/cm^2^; Millipore, MO, USA) with 20% FBS EGM-2 medium (Lonza, MD, USA). The medium was changed after 4 days, and early EPCs developed an elongated spindle-shaped morphology after 7 days of culture. Then, the medium was changed every 2 days. After 2–3 weeks, ECFCs were identified by their cobblestone-like morphology and expression of surface markers including CD31, KDR, and vWF. Thereafter, ECFC colonies were trypsinized and cultured on fibronectin pre-coated wells or plates (2 × 10^4^/cm^2^) for further experiments.

### Characterization of early EPCs and ECFCs

Fluorescence-activated cell sorter (FACS) was used to characterize the adherent cell population via antibodies against CD34 (BD Biosciences, NJ, USA), kinase insert domain receptor (KDR)/VEGF receptor 2 (BD Biosciences, NJ, USA), VE-cadherin (BD Biosciences, NJ, USA), AC133 (CD133; BD Biosciences, NJ, USA), platelet-endothelial cell adhesion molecule-1 (CD31; BD Biosciences, NJ, USA) and CD45 (Biolegend, CA, USA). Flow cytometry was performed using a FACS Canto flow cytometer (BD Biosciences, NJ, USA).

### miRNA quantitative real-time RT-PCR analysis

Total RNA was isolated from early EPCs and ECFCs using TRIzol Reagent (Thermo Scientific, MA, USA), and RNA was converted to cDNA using a Synthesis Kit (Thermo Scientific, MA, USA). qRT-PCR was performed using a Roche Light Cycler 480 (Roche, Switzerland) and miRNA qPCR Quantitation Kit (GenePharma, Shanghai, China) according to the manufacturer’s instructions. U6 level was used for normalization. PCR primers (forward and reverse, respectively) were as follows: has-miR-150, 5′-GTCGGGGGAGTGTTGCCTCCTCCCCACC-3′ and 5′-GGTGGGGAGGAGGCAACACTCCCCCGAC-3′; U6, 5′-GCTTCGGCAGCACATATACTAAAAT-3′ and 5′-CGCTTCACGAATTTGCGTGTCAT-3′; β-actin, 5′-ACATCCGCAAAGACCTGTAC-3′ and 5′-GCCATGCCAATCTCATCTTG-3′; and c-Myb, 5′-TGCCTCAAATTGGACTTTGG-3′ and 5′-GATTGAAATTCTGTGTAACTGC-3′.

### Agomir and antagomir transfection

To overexpress miR-150 in early EPCs, miR-150 agomir (GenePharma, Shanghai, China) was transfected using Lipofectamine 3000 (Invitrogen, CA, USA). Meanwhile, to knockdown miR-150 expression in ECFCs, miR-150 antagomir was transfected according to the manufacturer’s instructions. Transfection efficacy was > 90%, based on the expression of a co-transgene, green fluorescent protein (GFP). The sequence of miR-150 agomir was as follows: 5′-UCUCCCAACCCUUGUACCAGUG-3′ (sense) and 5′-CUGGUACAAGGGUUGGGAGAUU-3′ (antisense); antagomir (sense) 5′-CACUGGUACAAGGGUUGGGAGA-3′. The target sequence of siRNA against c-Myb (sense + loop + antisense) was 5′-GGTGGAACAGAATGGAACATTGAAGAAG TGTTCCATTCTGTTCCACC TT-3′.

### Fluorescence-activated cell sorter analysis

Using antibodies against CD34, VEGFR2, VE-cadherin, AC133, CD31, and CD45, stained cells were analyzed by FACS. Briefly, cells were resuspended at 1 × 10^6^ cells/ml in PBS and incubated 30 min with 5 μg/ml antibody on ice in the dark. Cells were washed twice with PBS and fixed with 1% paraformaldehyde for 10 min at 4 °C. Cells were washed and resuspended at 1 × 10^6^ cells/ml in PBS for FACS.

### Tube formation assay

Matrigel (BD Biosciences, NJ, USA) basement membrane matrix was added to a 24-well plate. After 1 h of incubation at 37 °C, 5 × 10^4^ early or ECFCs were seeded into the plate with EGM-2MV media. Twenty-four hours later, six representative fields were imaged, and the average of the total area of complete tube formation measured by cells/unit area was accomplished with the ImageJ software (MediaCybernetics, MD, USA). The tube length obtained from miR-150 agomir-transfected ECFCs was set as 100.

### In vivo Matrigel plug assay

Experiments were conducted in accordance with the institutional guidelines and approved by the Nanjing Drum Tower Hospital Institutional Animal Care and Use Committee. Specific pathogen-free 6-week-old nude mice were injected subcutaneously on the abdominal midline with 0.6 ml of Matrigel containing EPCs (5 × 10^6^ cells). After 1 week, the solid gel plug was removed and fixed in methanol overnight (4 °C) then embedded in paraffin, and 5-μm-thick sections were cut and stained with hematoxylin-eosin (HE). The vessels were counted using the ImageJ software.

### ELISA assay

Culture supernatant cytokines were quantified using the Quantikine human VEGF and IL-8 ELISA kit (R&D, MN, USA) according to the manufacturer’s instructions. To assess the secretion of VEGF and IL-8 after culturing EPCs, transfected EPCs and early or ECFCs were added to the ELISA to maximize low signals. Optical densities were measured at 450 nm, and 595 nm was used as the reference wavelength.

### Proliferation assay

Early EPCs, ECFCs, and transfected EPCs (1 × 10^3^ cells) were respectively seeded to each well of a 96-well plate in a final volume of 200 μl/well EGM-2 medium for proliferation assessment. After 48 h of incubation, cell proliferation was evaluated using the Cell Counting Kit-8 (Dojindo, Kumamoto, Japan). All experiments were performed in triplicate.

### Luciferase assays

The pMIR-c-Myb-3′UTR plasmid containing the putative binding site of c-Myb 3′UTR downstream of the firefly luciferase gene was generated by cloning and inserting of a mutated sequence located at 3′UTR into the SpeI and HindIII sites of the pMIR-REPORT Luciferase vector (Ambion, TX, USA). For the 3′UTR reporter assays, miRNA agomir and reporter plasmids were co-transfected by using Lipofectamine 3000 reagent (Invitrogen, CA, USA) into 293T cells and analyzed by the measurement of the ratio between firefly and Renilla luciferase activities.

### Western blot analysis

Total proteins were extracted from EPCs using RIPA buffer and high-speed centrifugation (12,000 rpm, 15 min) and quantified by the bicinchoninic acid method. Equal amounts of proteins were separated by SDS-PAGE electrophoresis and transferred to the PVDF membranes, which were blocked with 5% non-fat milk TBST and incubated with antibodies for c-Myb, total Akt, p-Akt, FOXO1, p-FOXO1 (Abcam, MA, USA), and β-actin (Sigma, MO, USA). After reaction with appropriate horseradish peroxidase-conjugated secondary antibodies, the protein bands were examined using Super Signal West Pico Chemiluminescent Substrate (Pierce, Rockford, IL) on X-ray film (Kodak, Tokyo, Japan).

### Vector construction, lentivirus production, and cells transduction

The lentiviral expression vector pGLV3-H1-GFP-Puro-hsa-miR-150 was constructed to stably express miR-150 in EPCs. 293T cells were co-transfected with pGLV3-H1-GFP-Puro vector or pGLV3-H1-GFP-Purolet-hsa-miR-150 plasmid using Lipofectamine 3000 (Invitrogen, CA, USA). Cells positive for GFP expression were detected via microscopy.

### Rat model of venous thrombosis

All procedures were approved by the Institutional Animal Care and Use Committee of Nanjing Drum Tower Hospital. Athymic nude rats (8–12 weeks; Charles River Laboratories, Beijing, China) were anesthetized by intraperitoneal injection of 7% pentobarbital and underwent midline laparotomy to dissect the inferior vena cava (IVC) from the aorta. IVC occlusion was achieved with 7-0 Prolene suture just below the renal vein. For complete blood flow obstruction, all side branches were ligated. Neurosurgical vascular clips were attached to the iliolumbar tributary veins for 15 min. The incision was closed, and the rats were allowed to recover after surgery. All rats were divided into five groups and received vehicle or cell transplantation (*n* = 10/group): (A) blank control group received 1 ml cell culture medium, (B) eEPC group (eEPC) received 1.0 × 10^6^ eEPCs, (C) eEPC/miR-150+ ECFCs group received 5.0 × 10^5^ eEPCs transfected with lentivirus particle of pGLV3-H1-GFP-Puro-hsa-miR-150 and 5.0 × 10^5^ ECFCs, (D) eEPC/NC+ ECFCs group received the same amount of EPCs transfected with lentivirus particle of pGLV3-H1-GFP-Puro vector, and (E) ECFCs group received 1.0 × 10^6^ ECFCs.

### Tissue harvest and histology

Seven days post-EPC or control medium injection, animals were sacrificed and IVC segments with thrombus were carefully harvested. Before weighing the thrombi, excess blood on the thrombi was removed by a filter paper. The thrombi were then treated with 4% paraformaldehyde overnight, and the samples were dehydrated using a graded ethanol series, treated by dimethylbenzene and paraffin-embedded. The 5-μm sections at 200 μm intervals were collected throughout the length of the thrombus samples. The sections underwent HE staining or immunohistochemistry.

### Digital subtraction angiography

Venography was performed via digital subtraction angiography (DSA) (GE Innova 3100; GE, MA, USA). Under general anesthesia, the tail vein was injected with a contrast medium using a custom-built, power micro-injector. A contrast medium was also injected into IVC to evaluate the recanalization and resolution of thrombus. DSA projections were acquired at 30 frames/s using a detector (944 × 704 px), 80 kV tube voltage, and 75 μA current. DSA sequences were recorded digitally in an Audio Video Interleave (AVI) movie file format, and files were imported using ImageJ and decomposed to single projections.

### Statistical analysis

All values are expressed as mean ± SD. Student’s *t* test was applied for two-group comparisons, while one-way ANOVA was used for comparing more than two groups. All statistical analyses were performed using SPSS version 21 (IBM, IL, USA). A two-tailed value of *P* < 0.05 was considered a significant difference.

## Results

### miR-150 promoted EPC differentiation

**Fig. 1:**
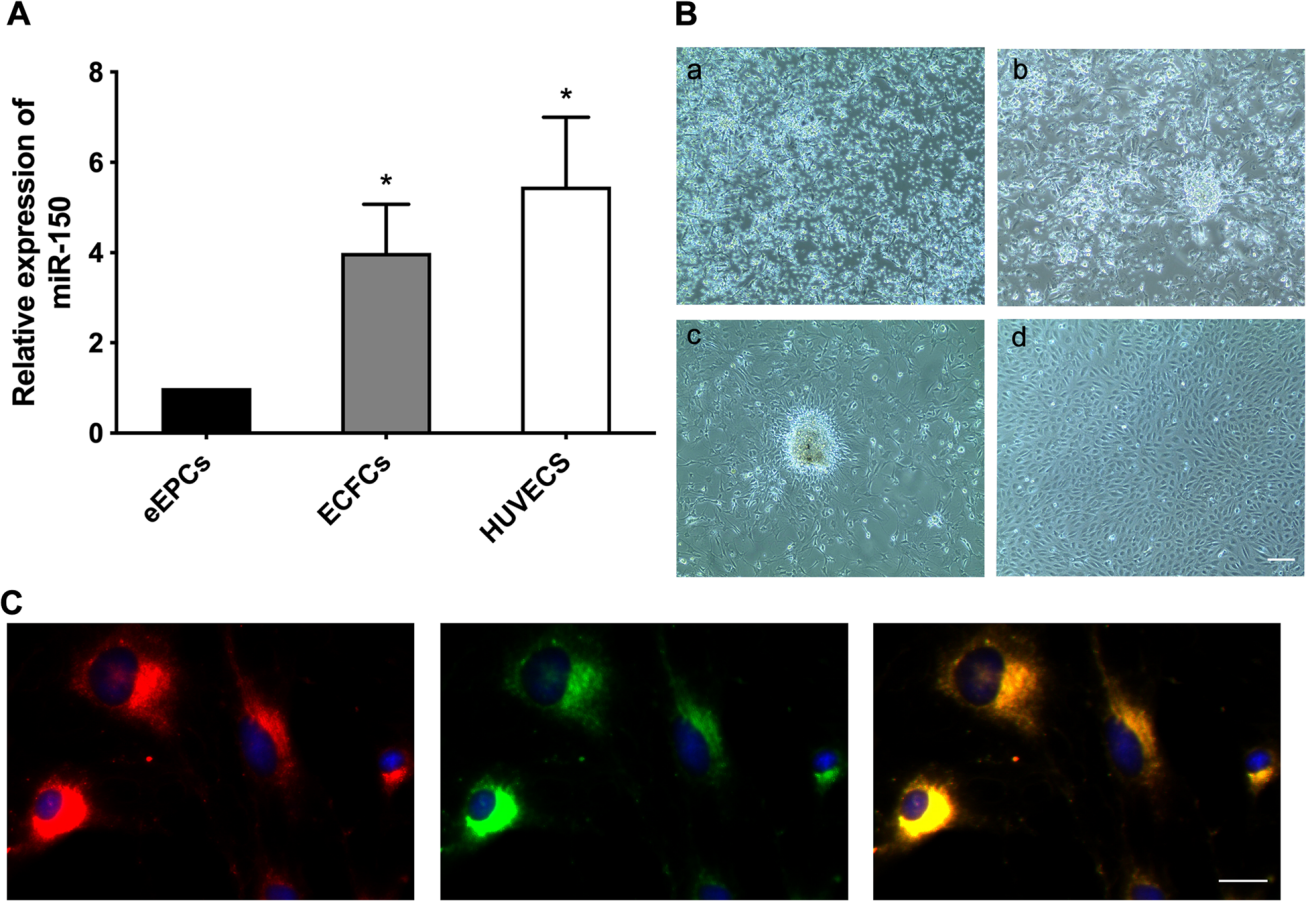
Characterization of eEPCs and ECFCs. **A** Endogenous expression of miR-150 measured by real-time RT-PCR in eEPCs, ECFCs, and HUVECs. **B** Morphological changes of EPCs. (a) Peripheral blood mononuclear cells showed multiple morphologies immediately after plating. (b) Five days after seeding, PBMCs formed a central cluster. (c) On day 12, EPCs exhibited a spindle shape, endothelial cell-like morphology. (d) Two weeks after plating, ECFCs grown to confluence exhibited a cobblestone appearance similar to HUVEC. **C** ECFCs were stained with Dil-ac-LDL (red) and lectin (green) and the merged image showed double staining of Dil-ac-LDL and FITC-UEA-1 (yellow)

miR-150 expression was first evaluated in eEPCs and ECFCs by qRT-PCR. We observed increased expression of miR-150 in ECFCs compared with eEPCs (Fig. 1A). The initially seeded cells showed multiple types of morphology (Fig. 1B (a)). During differentiation, PBMCs formed a central cluster on day 5 (Fig. 1B (b)) and exhibited a spindle-shape, endothelial cell-like morphology for 12 days (Fig. 1B (c)). After 14 days of culture, a cobblestone appearance similar to HUVEC was observed (Fig. 1B (d)). Further, EPCs were characterized as adherent cells positive for DiI-labeled acetylated low-density lipoprotein (Di-ac-LDL) uptake and lectin binding (Fig. 1C).

**Fig. 2:**
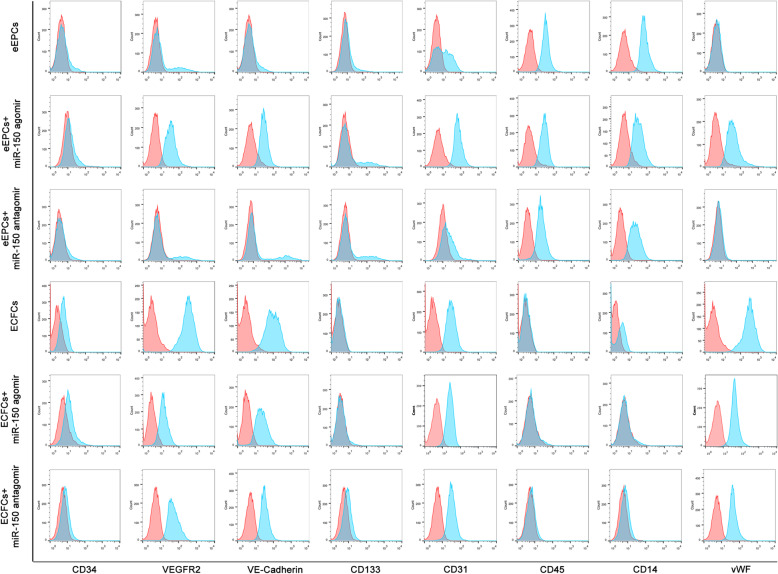
Flow cytometry was performed to determine the cell markers (CD34, VEGFR2, VE-cadherin, CD133, CD31, CD45, CD14, and vWF) of eEPCs, eEPCs transfected with miR-150 agomir or antagomir, ECFCs, and ECFCs transfected with miR-150 agomir or antagomir

To define how miR-150 influences EPC differentiation, we overexpressed or downregulated miR-150 using an agomir or antagomir, respectively. Flow cytometry showed that eEPCs expressed CD45, CD14, CD34, CD31, and CD133 but weakly expressed VEGFR2 and vWF. ECFCs, however, showed increased expression of all endothelial markers such as VEGFR2, VE-Cad, vWF, and CD31, whose expression is shared by monocytes. Interestingly, miR-150 upregulation in eEPCs promoted strong expression of VEGFR2, VE-Cad, and vWF, whereas the expression of pan-leukocyte marker CD45 and monocytes/macrophages marker CD14 decreased. Meanwhile, the downregulation of miR-150 in eEPCs did not change the expression level in most surface markers. Furthermore, transient inhibition of miR-150 with an antagomir induced decreased ECFC expression of CD34, CD31, and VE-Cad compared with that of non-transfected ECFCs, while increased miR-150 expression maintained the high expression of endothelial markers such as VEGFR2, CD31, and vWF (Fig. 2).

### miR-150 regulated early and ECFC function

**Fig. 3:**
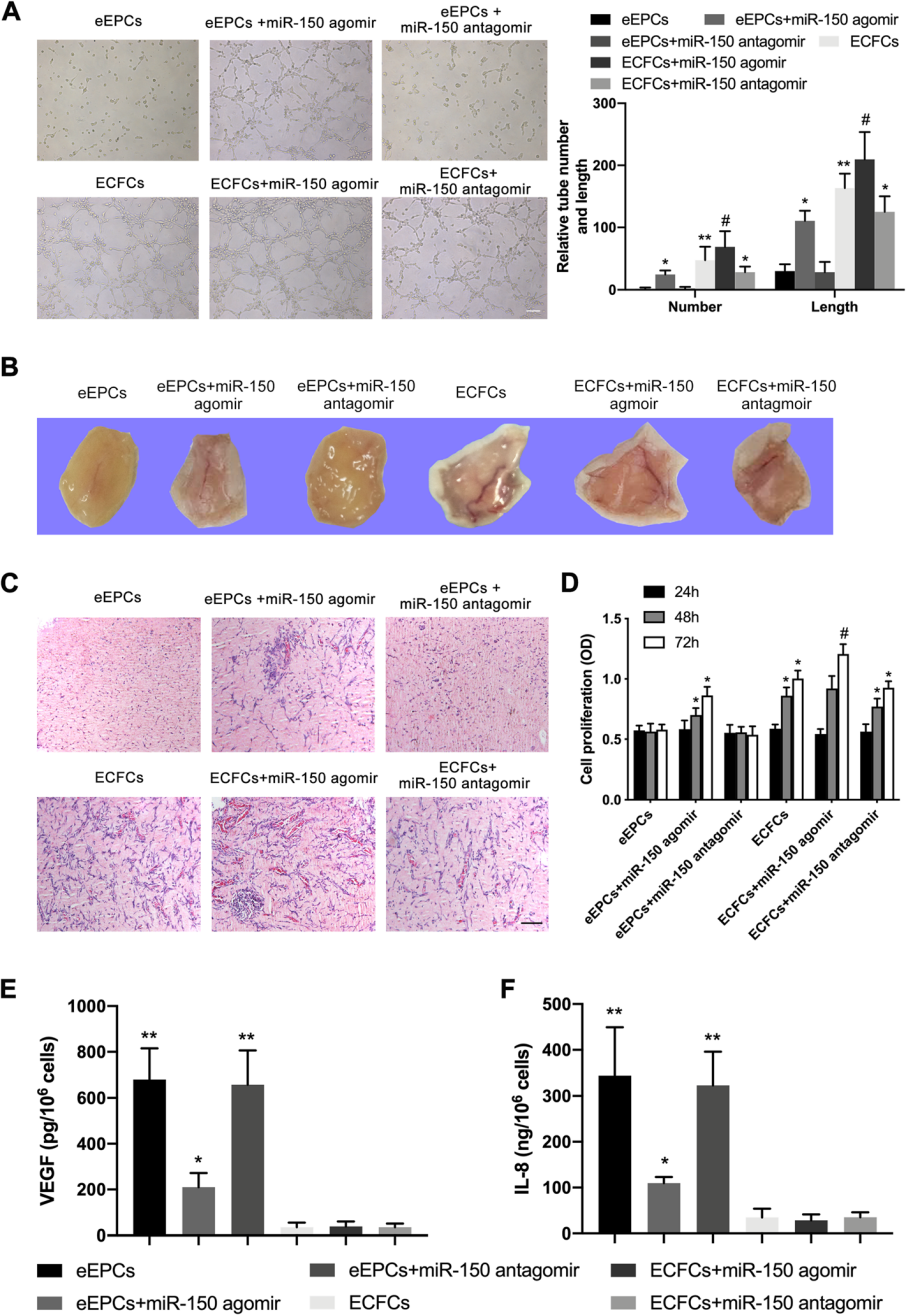
miR-150 regulates the function of early EPCs and ECFCs. **a** miR-150 were regulated in eEPCs and ECFCs. Then, angiogenetic capacity was examined in eEPCs, eEPCs transfected with miR-150 agomir or antagomir, ECFCs, and ECFCs transfected with miR-150 agomir or antagomir. Representative images (upper) and statistical analysis of three independent experiments (lower) are shown. **b** eEPCs transfected with miR-150 agomir or antagomir and ECFCs transfected with miR-150 agomir or antagomir were mixed with Matrigel and then injected subcutaneously on nude mice. The gel plug was removed after 1 week for the analysis of angiogenetic intensity. **c** The gel plugs were texted with HE stain (× 200). **d** CCK-8 assay was conducted to test the proliferation capacity in different groups (eEPCs, eEPCs+miR-150 agomir, eEPCs+miR-150 agomir, ECFCs and ECFCs+miR-150 agomir, ECFCs+miR-150 antagomir). **e** Concentrations of IL-8 and VEGF were measured in supernatant among different groups (eEPCs, eEPCs+miR-150 agomir, eEPCs+miR-150 agomir, ECFCs and ECFCs+miR-150 agomir, ECFCs+miR-150 antagomir). (*P < 0.05 vs. eEPCs; **P < 0.01 vs. eEPCs; ^#^*P* < 0.05 vs. ECFCs)

As miR-150 levels were significantly different between eEPCs and ECFCs, we hypothesized that differences in gene expression may lead to the functional differences between the two cell types. Overexpression of miR-150 in eEPCs increased EPC formation of tube-like structures compared to eEPCs (Fig. 3a). This phenomenon was consistent with observations from an in vivo Matrigel plug assay (Fig. 3b, c). Furthermore, miR-150 agomir increased the eEPC proliferation compared to control (Fig. 3d). In addition, miR-150 downregulation in ECFCs reduced angiogenesis, and upregulation of miR-150 further promoted angiogenesis and proliferation in ECFCs (Fig. 3a, d). Following an ELISA to test VEGF and IL-8 secretion by EPCs, results showed miR-150 downregulation reduced VEGF and IL-8 production in eEPCs (Fig. 3e, f). Meanwhile, both inhibition and promotion of miR-150 in ECFCs did not influence supernatant VEGF and IL-8 concentrations (Fig. 3e, f).

### miR-150 regulated the function of early EPCs and ECFCs via the Akt/FOXO1 pathway

**Fig. 4:**
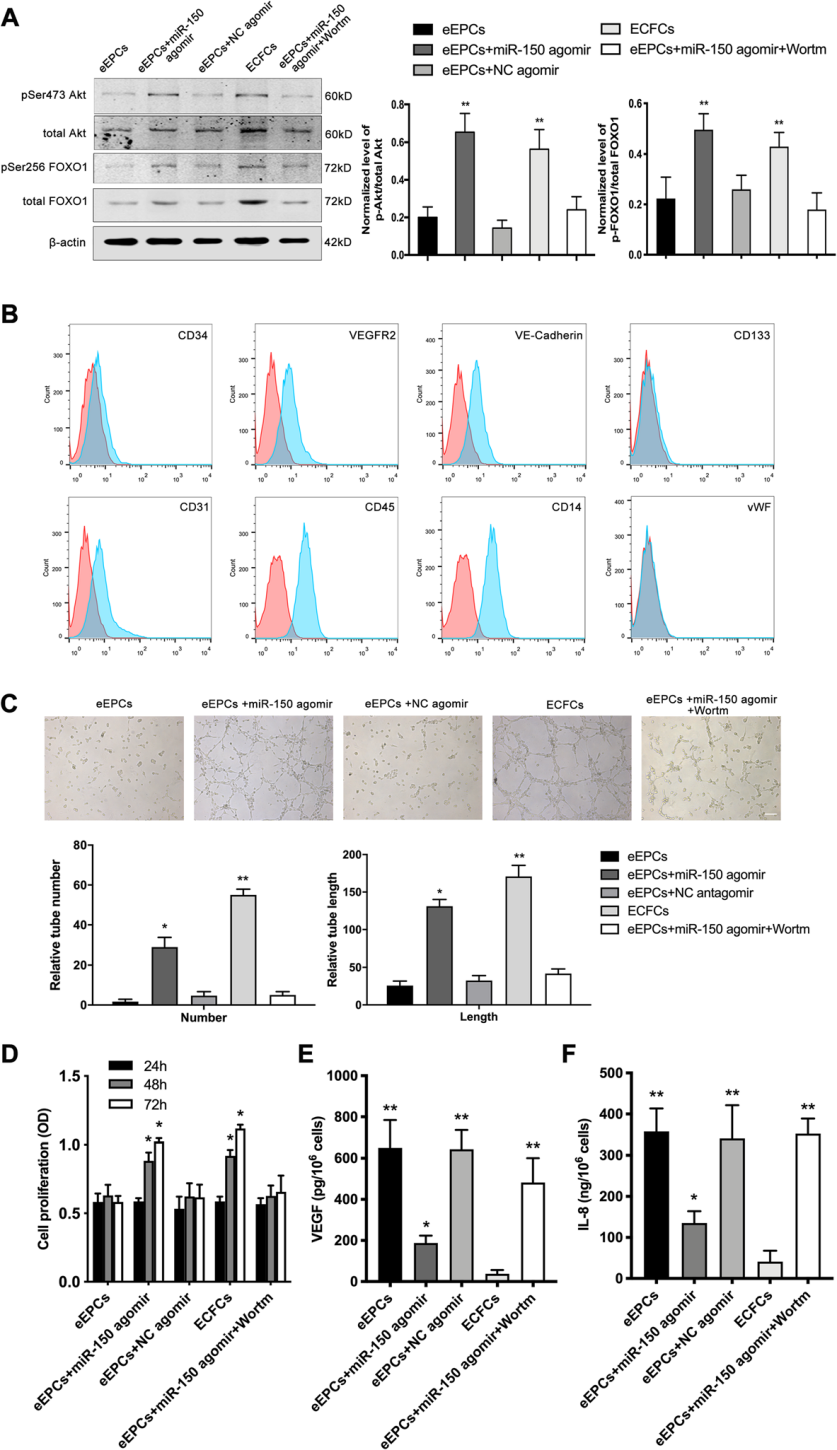
Effect of miR-150 regulates the function of early EPCs and late EPCs via the Akt/FOXO1 pathway. (**a**) Effect of miR-150 on the Akt/FOXO1 pathway, determined by Western blot 72 h after transfection. A representative blot was shown. **b** Cell surface markers (CD34, VEGFR2, VE-cadherin, CD133, CD31, CD45, CD14, and vWF) were measured in eEPCs with treated miR-150 agomir and wortmannin via flow cytometry. **c** Tube formation assay showed the change of angiogenetic capacity in the presence of wortmannin. Representative images (upper) and statistical analysis of three independent experiments (lower) are shown. **d**, **e** Proliferation capacity and cytokine concentration were tested among different groups (eEPCs, eEPCs+miR-150 agomir, eEPCs+NC agomir, ECFCs and eEPCs+miR-150 agomir+Wortm). (*P < 0.05 vs. eEPCs; **P < 0.01 vs. eEPCs)

Previous studies have reported that the Akt pathway is important in EPC differentiation [26–28]. We therefore analyzed the effect of miR-150 treatment on Akt signaling. Additional evidence demonstrated that Forkhead box-containing protein (FOXO) was downregulated through phosphorylation during EPC differentiation [28]. As a downstream target of Akt activity, we explored FOXO1 expression and phosphorylation (p-FOXO1^S256^). Western blot analysis showed Akt protein expression increased during differentiation, and miR-150 upregulation in eEPCs increased Akt phosphorylation at Ser473. Consistent with these observations, phosphorylation of FOXO1 at Ser256 also increased in eEPCs transfected with miR-150 (Fig. 4a). To confirm the role of Akt signaling in miR-150-dependent endothelial differentiation, we incubated miR-150 transfected eEPCs in the presence of PI3K inhibitor wortmannin. As shown in Fig. 4a, Akt pathway inhibition by wortmannin reduced Akt and FOXO1 phosphorylation. Consistently, FACS analysis revealed that wortmannin effectively reduced endothelial markers compared with miR-150 agomir (Fig. 4b). Surface antigens expressed are listed in Table 1. Furthermore, wortmannin reduced eEPCs’ ability to form tube-like structures when transfected with miR-150 agomir compared to control (Fig. 4c). Proliferation assay and ELISA also indicated that wortmannin reverted the effect of miR-150 in eEPCs (Fig. 4d, e). Taken together, these results demonstrated that miR-150 expression and the Akt/FOXO1 pathway are involved in the regulation of function in eEPCs and ECFCs.

**Table 1:** Cell surface antigen expression

Antigen	eEPCs (%)	eEPCs+agomir150 (%)	eEPCs+NC agomir (%)	ECFCs (%)	eEPCs+agomir150+wortm (%)
CD34	9.22	12.60	8.53	19.60	11.10
VEGFR2	19.10	83.20	17.20	99.30	35.30
VE-Cad	8.84	52.10	10.80	95	31.80
CD133	7.85	2.79	6.74	1.47	4.35
CD31	36.10	92.60	42.35	97.30	26.40
CD45	92.20	59.40	93.70	1.90	92
CD14	93.40	48	90.80	0.26	92.30
vWF	1.67	61.80	2.35	99.10	2.37

### Effect of miR-150 during EPC differentiation was mediated by targeting c-Myb

**Fig. 5:**
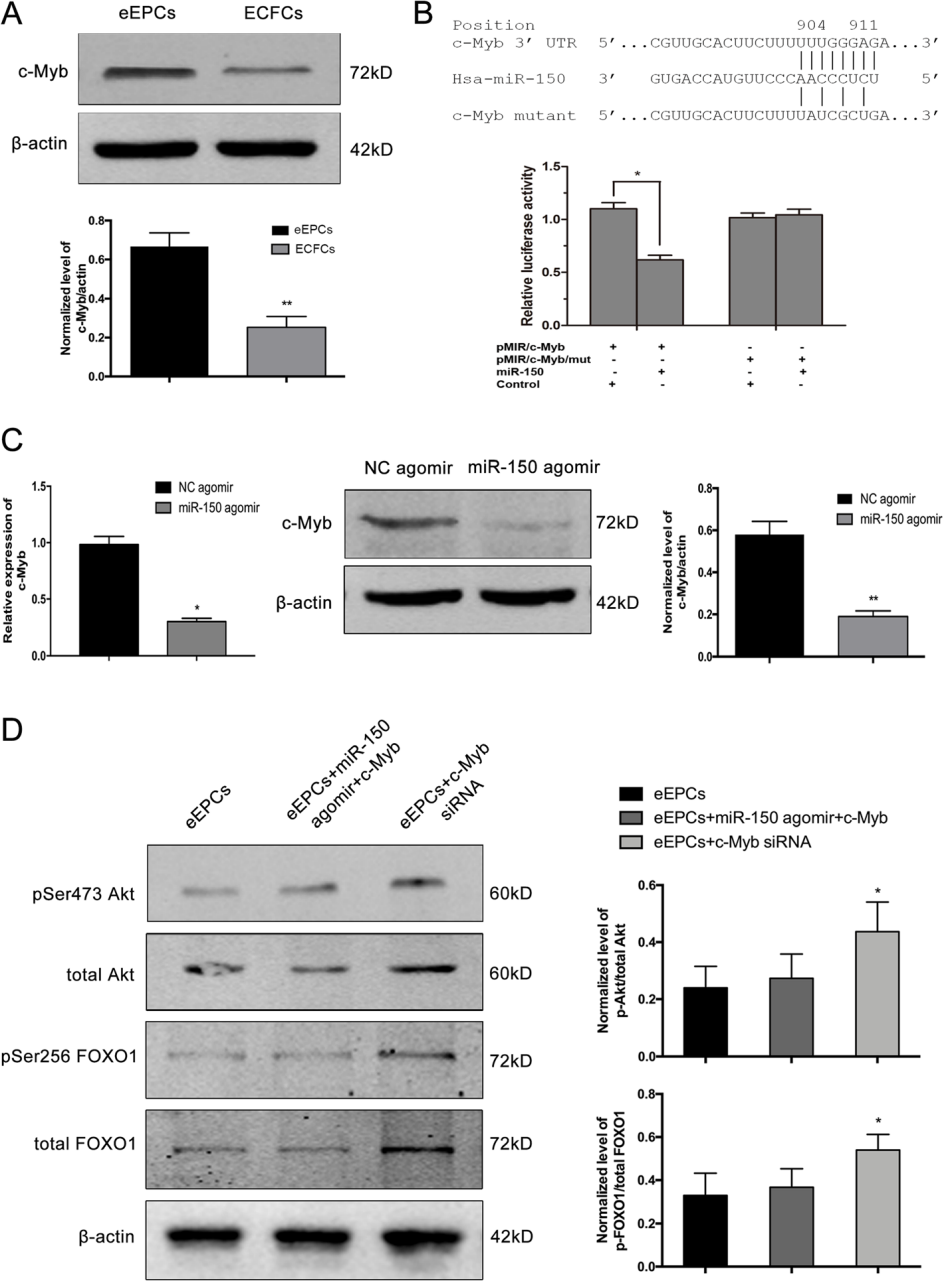
Effect of miR-150 during EPC differentiation is mediated by targeting c-Myb. **a** c-Myb protein level in eEPCs and ECFCs, measured by Western blot. **b** Dual-luciferase reporter assay was performed on 293T cells. Schematic graph of the constructed reporter plasmid containing putative binding sites of miR-150 in the c-Myb 3′UTR. c-Myb 3′UTR mut indicates mutation in the miR-150 binding site (upper). Relative luciferase activity of c-Myb 3′UTR decreased as compared to mutation control (lower). **c** The effect of miR-150 on c-Myb expression was confirmed by real-time RT-PCR (left) and Western blot (right). **d** Western blot analysis revealed the effect of miR-150 on the Akt/FOXO1 pathway

c-Myb plays an essential role in regulating hematopoietic cell differentiation and may be directly regulated by miR-150, as forced miR-150 expression decreased c-Myb in rat EPCs [24, 29]. Therefore, we hypothesized that c-Myb contributes to the endothelial differentiation of EPCs. Western blot results showed diminished expression of c-Myb in ECFCs compared to eEPCs (Fig. 5a). Results from our luciferase report assay showed decreased luciferase activity in a luciferase report vector within a 340-bp region of 3′UTR c-Myb (Fig. 5b). In addition, we transfected miR-150 agomir into eEPCs and found that miR-150 upregulation significantly decreased c-Myb expression at both mRNA and protein levels (Fig. 5c).

To further explore the role of c-Myb in miR-150-dependent endothelial differentiation, we evaluated Akt/FOXO1 signaling in eEPCs transfected with miR-150 plasmid, vector control, or c-Myb siRNA. We found that c-Myb blocked miR-150 effects on Akt/FOXO1 signaling, while c-Myb downregulation activated this pathway (Fig. 5d). Therefore, our data indicate that miR-150 modified c-Myb expression, subsequently regulating Akt/FOXO1 signaling and stimulating EPC differentiation.

### Coinjection of ECFCs and miR-150-transfected eEPCs promoted thrombus recanalization and resolution

EPCs are increasingly recognized as a promising therapeutic for DVT-related thrombus resolution due to their capacity for differentiating into mature endothelial cells and secretion of angiogenic growth factors and cytokines. miR-150 has been shown to promote EPC recruitment to ischemic tissue and contribute to thrombus resolution and neovascularization. Meanwhile, mixed transplantation of eEPCs and ECFCs resulted in synergistic neovascularization through cytokines and MMPs. As such, we hypothesized that coinjection of eEPCs and ECFCs could promote thrombus resolution. Furthermore, miR-150 may participate in this process via regulating eEPC differentiation.

**Fig. 6:**
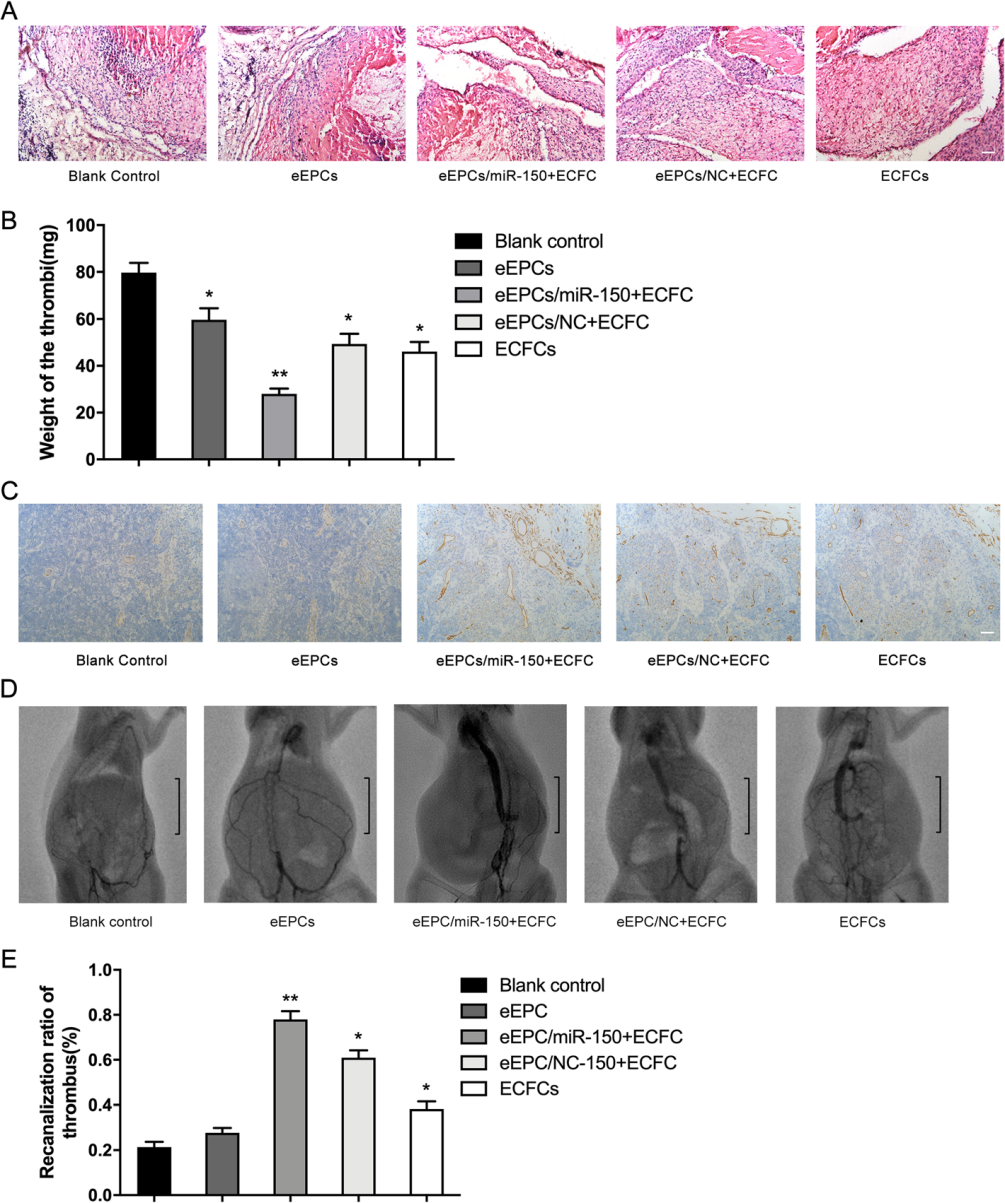
Therapeutic role of miR-150 in deep venous thrombosis. **a** HE staining showed that coinjection of eEPCs transfected with miR-150 and ECFCs promoted thrombus recanalization and resolution (× 400). **b** Weight of thrombus was tested at 7 days after transplantation. **c** Immunohistochemistry revealed more endothelial-specific marker CD34 expression in the EPCs/miR-150 plus ECFCs group as compared to other groups (× 400). **d** DSA images showed thrombus resolution in the inferior vena cava (IVC) in different groups (blank control, eEPCs, eEPCs transfected with miR-150 agomir plus ECFCs, and eEPCs transfected with NC agomir plus ECFCs). Black area in IVC indicated thrombus resolution. **e** The recanalization of thrombus was quantified with ImageJ software by the area ratio of contrast agent in vascular with thrombosis. (*P < 0.05 vs. blank control; **P < 0.01 vs. blank control)

HE staining showed that endothelial cells, monocytes, and neotrophil granulocytes stained with hyperchromatic nuclei entered the thrombus in our DVT model. More channel structures were present in both the eEPCs/miR-150 plus ECFCs and eEPCs/NC plus ECFCs groups than the control group at day 7. However, larger channels were found in the EPCs/miR-150 plus ECFCs group compared with the eEPCs/NC plus ECFCs group (Fig. 6a). Furthermore, thrombus weight was reduced by eEPCs/miR-150 plus ECFCs compared to other groups (Fig. 6b). Harvested thrombi were stained for CD34, showing more endothelial-specific marker expression in the EPCs/miR-150 plus ECFCs group (Fig. 6c).

We then evaluated thrombus recanalization and resolution in vivo by using DSA. DSA is useful in diagnosing and treating venous and arterial occlusion. Results showed that rats transplanted with eEPCs/miR-150 plus ECFCs showed significantly increased thrombus recanalization and resolution compared to other groups (Fig. 6d, e).

## Discussion

Stem cells and progenitor cells have become hotspot in various research fields with their characteristics of self-renewal and capacity to differentiate into specialized cell types, which are believed to be the novel therapy for various diseases [30, 31]. EPCs can exhibit different morphology, proliferation rate, vasculogenic potential, and survival features. In addition, early EPCs differ from ECFCs in gene expression profiles. Gulati et al. [32] reported eEPCs expressed endothelial antigens CD31, KDR, and Tie-2, but not VE-cadherin, while the endothelial surface antigen profile of ECFCs was more distinct. Another research found that ECFCs exhibited strong expression of all endothelial genes including VE-cadherin, Flt-1, KDR, e-NOS, and vWF at the same level as HUVECs [7]. In our study, we established that eEPCs expressed surface antigens including CD45, CD14, CD34, CD31, and CD133 and weak expression of endothelial markers VEGFR2 and vWF. However, ECFCs showed increased expression of all endothelial genes including VEGFR2, VE-Cad, and vWF. Additionally, CD31 levels were also elevated. Furthermore, it was reported that eEPCs exhibit a short lifespan of 3–4 weeks compared to the longer lifespan and enhanced proliferative ability of ECFCs [7]. We confirmed this difference between the two EPCs via proliferation assay. Currently, there are strong opinions concerning therapeutic angiogenesis with endothelial progenitor cells (EPCs). It is believed that ECFC angiogenic capacity is stronger than eEPCs, which was supported by our findings. The two types of EPCs also showed different Il-8 and VEGF secretion levels. IL-8 and VEGF were highly expressed by eEPCs in vitro. The effects of eEPCs on increased migration and tube formation of ECFCs may be explained by these results.

miR-150 is enriched in monocytes and is critical for cell proliferation, differentiation, and embryonic development [24, 33], and miR-150 promotes EPC motility in vitro [24]. We found that miR-150 was expressed differentially in eEPCs and ECFCs, suggesting an important role for miR-150 during EPC differentiation. Forced expression of miR-150 upregulated endothelial marker expression and increased tube formation capacity, as well as reduced proliferation and secretion effects. c-Myb is important for cell proliferation, differentiation, and survival [34], and secreted miR-150 involvement in migration and angiogenic regulation HMEC-1 cells via targeting c-Myb has been demonstrated [35]. miR-150/c-Myb interaction has a potentially important role in B cell differentiation [13]. In line with previous studies, bioinformatics and luciferase reporter assay analyses in our study revealed that miR-150 directly regulated c-Myb expression. Furthermore, we observed that miR-150 is involved in EPC differentiation via targeting c-Myb by genetically manipulating its expression in gain- and loss-of-function experiments. These results led to a miR-150-dependent downregulation of c-Myb levels, thus promoting EPC differentiation.

Although several classic signaling pathways were shown to participate in regulating stem cell function [36, 37], Akt signaling has also been shown to be important in EPC differentiation [26, 27]. Marchetti et al. [38] found that hyperglycemia impaired EPC differentiation, and this process could be restored by benfotiamine administration via modulation of Akt/FOXO1 activity. Evidence suggests Akt/FOXO3a signaling contributes to EPC differentiation [28]. In our system, enhanced miR-150 expression on eEPCs increased Akt phosphorylation at Ser473 and phosphorylation of FOXO1 at Ser256, signifying a potential contribution of Akt/FOXO1 signaling to EPC functional differentiation. To further verify this association, wortmannin treatment reverted miR-150 effects in eEPCs. We also established that the application of wortmannin blunted Akt and FOXO1 phosphorylation. Moreover, c-Myb inhibition activated Akt/FOXO1 activity while c-Myb upregulation blocked miR-150 effects on this pathway. Taken together, our results shed new light on the role of miR-150 on EPC differentiation and function via regulating both c-Myb expression and Akt/FOXO1 signaling.

Venous thrombi are characterized by a laminar structure with two components including aggregated platelets and red blood cells that form a plug and a mesh of cross-linked fibrin protein [39]. Thrombus resolution is complex and requires the orchestration of endothelial cells, inflammatory cells, and fibroblasts [40]. However, natural thrombus resolution is slow, giving rise to chronic debilitating clinical complications. Standard DVT anticoagulation therapy prevents venous propagation but has limited efficacy in removing the existing thrombus. Besides, the process of thrombus resolution resembles wound healing and other previous study demonstrated stem cells and microRNA were involved in this process [41]. Emerging evidence suggests that stem and progenitor cells contribute to tissue vascularization, and EPCs are a type of progenitor cell with potential clinical value for facilitating venous thrombus resolution. Several studies have reported that circulating EPCs could promote thrombus recanalization by restoring impaired endothelium and accelerating neovascularization [40, 42]. Yoon et al. confirmed that transplanting mixed EPCs results in synergistic augmentation of angiogenesis in athymic nude mice with hind limb ischemia [10]. Considering the differing function and roles of these cell types in neovascularization, it is useful to identify factors that promote differentiation of the progenitor cells. In our present study, we verified that miR-150 played an important role in regulating EPC differentiation. More importantly, the aberrant overexpression of miR-150 in eEPCs enhances thrombus recanalization and resolution. This result can be explained by different functions exhibited by these two cell types. The upregulation of miR-150 promoted eEPC differentiation and increased angiogenic potential. Additionally, eEPCs contributed to neovascularization via growth factor and cytokine secretion, such as IL-8 and VEGF.

## Conclusions

In conclusion, we established that miR-150 influenced EPC differentiation by inhibition of c-Myb and activation of Akt/FOXO1 signaling and therefore modulated the function of eEPCs and ECFCs ex vivo. Moreover, the coinjection of miR-150-transfected eEPCs with ECFCs promoted thrombus recanalization and resolution in vivo. These data suggest that miR-150 might be a valuable therapeutic target in the clinical treatment of thrombus.

## Data Availability

All data generated or analyzed during this study are included in this published article.

## Abbreviations

CD: Cluster of differentiation
Di-ac-LDL: DiI-labeled acetylated low-density lipoprotein
DSA: Digital subtraction angiography
DVT: Deep venous thrombosis
eEPCs: Early endothelial progenitor cells
EPCs: Endothelial progenitor cells
FACS: Fluorescence-activated cell sorter
FOXO1: Forkhead box protein O1
GFP: Green fluorescent protein
HE: Hematoxylin-eosin
IVC: Inferior vena cava
KDR: Kinase insert domain receptor
lEPCs: Late endothelial progenitor cells
ECFCs: Endothelial colony-forming cells
miR: MicroRNA
PBMCs: Peripheral blood mononuclear cells
PTS: Post-thrombotic syndrome
UTRs: Untranslated regions
VEGF: Vascular endothelial growth factor
